# Rhizobial–Host Interactions and Symbiotic Nitrogen Fixation in Legume Crops Toward Agriculture Sustainability

**DOI:** 10.3389/fmicb.2021.669404

**Published:** 2021-06-11

**Authors:** Ravinder K. Goyal, Autar K. Mattoo, Maria Augusta Schmidt

**Affiliations:** ^1^Agriculture and Agri-Food Canada, Lacombe Research and Development Centre, Lacombe, AB, Canada; ^2^Sustainable Agricultural Systems Laboratory, Agricultural Research Service, United States Department of Agriculture, Beltsville Agricultural Research Center, Beltsville, MD, United States

**Keywords:** BNF, SNF, QTLs, rhizobia, symbiosis, host-specificity, environmental factors

## Abstract

Symbiotic nitrogen fixation (SNF) process makes legume crops self-sufficient in nitrogen (N) in sharp contrast to cereal crops that require an external input by N-fertilizers. Since the latter process in cereal crops results in a huge quantity of greenhouse gas emission, the legume production systems are considered efficient and important for sustainable agriculture and climate preservation. Despite benefits of SNF, and the fact that chemical N-fertilizers cause N-pollution of the ecosystems, the focus on improving SNF efficiency in legumes did not become a breeder’s priority. The size and stability of heritable effects under different environment conditions weigh significantly on any trait useful in breeding strategies. Here we review the challenges and progress made toward decoding the heritable components of SNF, which is considerably more complex than other crop allelic traits since the process involves genetic elements of both the host and the symbiotic rhizobial species. SNF-efficient rhizobial species designed based on the genetics of the host and its symbiotic partner face the test of a unique microbiome for its success and productivity. The progress made thus far in commercial legume crops with relevance to the dynamics of host–rhizobia interaction, environmental impact on rhizobial performance challenges, and what collectively determines the SNF efficiency under field conditions are also reviewed here.

## Introduction

**FIGURE 1:**
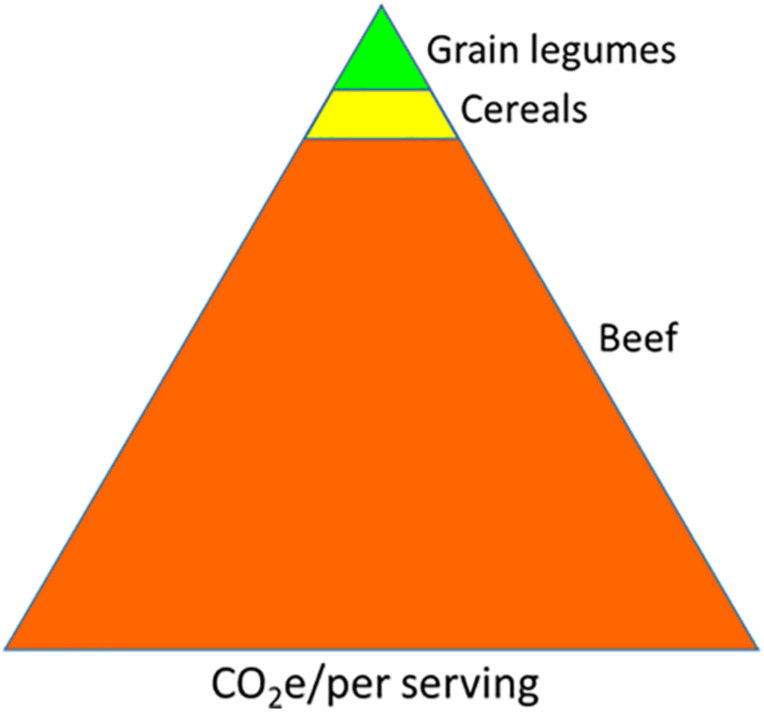
Comparison of carbon footprint generated by per unit serving of different food types. The areas in the triangles are approximate to carbon footprint values (http://css.umich.edu/factsheets/carbon-footprint-factsheet).

Biological nitrogen fixation (BNF) is the largest nitrogen (N) input pathway in the ecosystem (Vitousek and Field, 2001). It is estimated that BNF generates approximately 200 Tg of organic N annually (Galloway et al., 2003; Peoples et al., 2009); thus, it can play an important role in climate preservation and agriculture sustainability. In legumes, a group of bacteria collectively referred to as rhizobia enzymatically convert atmospheric nitrogen into organic form through a symbiotic process (symbiotic N fixation, SNF) in the host roots. The grain legumes account for 27% of the world crop production and are only next to cereals. Globally, 60% of the total human protein requirement is met through plant-based proteins, of which legumes contribute to about 50% (Smýkal et al., 2015; ref. in Leinonen et al., 2019). An assessment of global protein sustainability favors a shift from animal-based to plant-based proteins (Chaudhary et al., 2018; Leinonen et al., 2019)^1^. The legumes are valuable in agriculture for two main reasons. One, the legume seeds are rich in protein content (20–25% of the dry weight) and help meet the daily protein requirement of a majority of the world population, especially for those who cannot afford to source it from animals. Second, the environmental cost of legume cultivation, calculated as life cycle assessment (LCA), is significantly lower compared to non-legume crops due to the former’s ability to fix their own nitrogen (MacWilliam et al., 2014; Romeiko, 2019). Despite the challenges of accuracy and precision, the LCA is calculated by computing greenhouse gas (GHG) emission of all operations associated with a crop production system and delivering a product to the consumer table (Caffrey and Veal, 2013). The carbon footprint comparison depicts the lowest environmental cost of legumes among important food types (Figure 1).

In order to emphasize the significance of legumes in agriculture sustainability, it is pertinent to consider the environmental consequences of synthetic N use. The annual demand of chemically synthesized nitrogen is projected at about 120 Tg (FAO, 2017), approximately 50% of which is required to fertilize wheat, maize, and rice (Ladha et al., 2016). The N-use efficiency for cereals is estimated to be about 33%; the remaining becomes either a source of aquatic systems pollution (Raun and Johnson, 1999) or a substrate for N_2_O or nitrous oxide (NO) gases in the environment through denitrification. According to the US Environmental Protection Agency (EPA), N_2_O has 294 times more GHG effect than carbon dioxide on a per unit mass basis. In addition, the process of nitrogen fertilizer synthesis uses fossil fuels as one of the energy sources. Depending on the type of the fossil fuel, it releases 1.10–3.37 t of CO_2_/ton fossil fuel burnt^2^. It has been predicted that 2% consumption of global energy by 2050 will be due to chemical synthesis of N fertilizers (Glendining et al., 2009). Approximately, one-third of total GHG emission occurs *via* the agriculture sector including crop cultivation and livestock (Gilbert, 2012). A large carbon footprint of agriculture along with the detrimental effects of runoff fertilizers puts a question mark on agriculture sustainability. The need for minimizing GHG emission and protecting the climate has been highlighted in a report by the Intergovernmental Panel on Climate Change (IPCC, see text footnote 2). In this connection, the legume production systems hold a great promise to reduce the agriculture carbon footprint by expanding the acreage under legumes and making the SNF process more efficient.

Low cost and easy accessibility of the synthetic N fertilizers obscured the importance of SNF. Most of the legume crop improvement strategies have focused on yield, disease resistance, and other agronomic traits. The availability of chemical fertilizers was one reason that interested breeders to utilize synthetic fertilizer, and the lack of interest in incorporating enhanced SNF efficiency as a trait in legume improvement programs was seen as far more complex since it involved a large array of gene medleys both in the host and in its symbiotic partner (Roy et al., 2020). Compared to an agronomic trait, the mapping of symbiosis-related genes on the chromosomes and understanding their heritable effect in breeding lines is far more challenging. Moreover, the stability of a heritable trait under different environmental conditions is very important, since it requires understanding and suitable deployment strategies for an optimal symbiotic efficiency. Introduction of novel and superior or genetically modified strains may face formidable challenges as the native strains are well-adapted to a specific niche. The development of elite rhizobial strains to maximize crop-specific symbiotic outcome is required which, however, has not received the needed attention (Sessitsch et al., 2002). In the recent past, an ecological relevance of rhizobia and the quest to gain in-depth knowledge of nitrogen fixation phenomenon have led to many advances. In this review, we bring together information relevant to the SNF as a breeding trait and the prominent factors that influence the symbiosis and N-fixing efficiency. This should help evoke more interest in filling the research gaps toward strengthening the symbiotic bond for a sustainable future.

## SNF-Associated QTLs and SNPs

Quantitative trait locus/loci (QTL) is the location of an individual locus or multiple loci in the genome that affects a quantitative trait. QTLs provide valuable information on the inheritance of linked traits which can be used to identify a candidate gene(s) associated with a phenotype. The DNA sequences linked with QTLs can be deployed as molecular markers for a phenotypic trait governed by them. Such molecular markers are being increasingly used in marker-assisted selection (MAS) in modern breeding programs (Kumar et al., 2017). Several successful examples of using QTLs to increase quality, yield, and disease resistance in cereal crops include rice (Ali et al., 2017), maize (Beyene et al., 2016), and wheat (Zhang et al., 2017). However, the use of QTLs for improving SNF has not gained prominence in legume breeding programs. Nodulation, which comprises nodule number, shape, size, fresh/dry weights, and type (determinate or indeterminate), along with the health of nodules, is an indicator of the N fixation efficiency and represents important quantitative SNF traits. The process of nodulation is tightly regulated by both the host and rhizobia genetics, collectively determining the output of fixed N (Wang et al., 2018; Ferguson et al., 2019). A high heritability of nodulation traits in a given environment is an indicator of traits controlled by genetic loci (Yang et al., 2019). A significant correlation is known between nodule number, nodule fresh or dry weight, and N fixed in a symbiotic process (Pereira et al., 1993; Pazdernik et al., 1996). A positive correlation has also been found between nodule dry weight/plant and the seed yield (Burias and Planchon, 1990). The SNF-QTLs have been identified in several legumes, including soybean, cowpea, pea, and common bean. Thus, identification of the genetic loci, QTLs, or markers associated with nodulation traits is useful in breeding legumes for an efficient N fixation and for improving yields. However, the focus or the progress has been conspicuously more in soybean and common bean than other commercial legumes (Table 1).

**TABLE 1:** QTLs and SNP loci information for the morphological traits associated with SNF.

Crop	Morphological trait	QTLs	References
Common bean (*Phaseolus vulgaris*)	Nodule number	4 QTLs	Nodari et al., 1993
Common bean	Nodule number	3 linkage groups under each of low and high nitrogen environments	Tsai et al., 1998
Common bean	Nodule number	5 QTLs in the presence of N	Souza et al., 2000
	Nodule number	3 QTLs in the absence of N	
Common bean	Ndfa	QTLs on Pv01, Pv04 and Pv10	Ramaekers et al., 2013
	Nodule dry weight	1 QTL on Pv03	
Dry bean (*Phaseolus vulgaris*)	Ndfa	1 QTL on Pv08	Farid, 2015
Common bean	Ndfa (in shoot and seed)	SNP loci on Pv03 and Pv09	Kamfwa et al., 2015
Common bean	%Ndfa	QTLs on Pv07 and Pv02	Diaz et al., 2017
Black bean (*Phaseolus vulgaris*)	Ndfa	1 QTL on Pv01	Heilig et al., 2017
Common bean	%Ndfa	3 QTLs on Pv01, Pv04 and Pv09	Kamfwa et al., 2019
	Total Ndfa	5 QTLs on Pv04, Pv06, Pv07, Pv09, and Pv11	
Soybean	Nodule number, nodule size, nodule dry matter, acetylene reduction	5 QTLs	Tanya et al., 2005
Soybean	Nodule number and nodule dry weight	2 QTLs	Nicolás et al., 2006
Soybean	Nodule number and nodule dry weight ratio or nodule dry weight per nodule, shoot dry weight	4 QTLs (B1/ *nn1-B* E/ *bnf3-E*, L/ *sdw2-L* and I/ *bnf4-II)*	Santos et al., 2013
Soybean	Symbiosis specificity	*Rj4* locus	Tang et al., 2014, Tang et al., 2016
Soybean	Nodule number	8 QTLs	Hwang et al., 2014
	Nodule size	7 QTLs	
	Individual nodule weight	6 QTLs	
	Total nodule weight	5 QTLs	
Soybean	Nodule fresh weight	1 QTL on Gm12	Muñoz et al., 2016
		1 QTL on Gm18	
Soybean	SNF traits and shoot dry weight	qBNF-C2 qBNF-O qBNF-B1	Yang et al., 2017
	Nodulation in the field (nodule number and weight)	*qBNF-11*	
Soybean	Number of large nodules and nodule weight	*GmINS1* gene	Li et al., 2018
Soybean	Average nodule dry weight	1 QTL on Gm13	Grunvald et al., 2018
Soybean	Symbiotic relationship (three nodules types) between indigenous rhizobia (*B. japonicum, B. elkanii, rhizobium sp*.) and soybean	24 QTLs (qBJ-11 to 14, qBJ-21 to 24, qBJ-31 and 32) spanning multiple linkage groups	Ramongolalaina et al., 2018
Soybean	Nodule size and nodule number	qBNF16	Yang et al., 2019
	Nodule size and nodule number	qBNF17	
Soybean	Nodule number, nodule fresh and dry weights	2 SNP loci on Gm17	Huo et al., 2019
	Ndfa (shoot)	3 SNP loci on Gm17	
Soybean	Nodule number and nodule dry weight	16 QTLs on eight chromosomes	Zhu et al., 2019
Soybean	Nodule number and nodule dry weight	2 QTLs on Gm19	Wang et al., 2020b
Soybean	*GmNNL1* gene is a negative regulator of nodulation	GmNNL1 locus	Zhang et al., 2021
Pea (*Pisum sativum* L.)	Nodule number	9 QTLs	Bourion et al., 2010
	Nodule size	8 QTLs	
	Nodule dry matter	4 QTLs	
	Relative part of the nodule dry matter	3 QTLs	
Cowpea (*Vigna unguiculata)*	Nodule color	QTL (Linkage group 4)	Ohlson et al., 2018
	Nodule number	QTL (Linkage group 4)	
	Nodule Fresh Weight	QTL (Linkage group 6)	
*Lotus japonicas*	Nitrogen fixation	*Sst1* gene	Krusell et al., 2005
*Lotus japonicus*	Nitrogen fixation symbiosis	*IGN1* gene	Kumagai et al., 2007
*Lotus japonicus*	Nodule number	1 QTL on Chr 3	Tominaga et al., 2012
	Nodule weight	5 QTLs on Chr 2,3,4 and 5	
	Acetylene reduction activity per plant	4 QTLs on Chr 2, 4 and 5	
	Acetylene reduction activity per nodule number	2 QTLs on Chr 2 and 4	
	Acetylene reduction activity per nodule weight	3 QTLs on Chr 3, 4 and 5	

Nitrogen fixation involves additional interactions with the strain and soil environment when compared to a plant agronomic trait and adds complexity in identifying SNF-QTLs in legumes. This could be a bottleneck for slow progress in the identification of QTLs in leguminous crops in comparison to cereal or other crops (Ali et al., 2017; Zhang et al., 2017; Fang et al., 2019). The nodule number was one of the early SNF-related traits shown to be controlled by QTLs in common bean under certain N environments (Nodari et al., 1993; Souza et al., 2000). Using simple sequence repeat markers, a linkage map was constructed in soybean and the markers were linked to fresh and dry nodule weights, nodule numbers, plant dry weight, and acetylene reduction activity corresponding to the N-fixation rate (Tanya et al., 2005; Nicolás et al., 2006). Some of the SNF-QTLs were genetically mapped in soybean, although the effect of these QTLs only ranged from 6.5 to 15.4% of total variation (Santos et al., 2013). Co-localization of QTLs for root growth and nodule traits and a significant positive relationship between nodule establishment and pea root growth are the breeding possibilities for these two important traits (Bourion et al., 2010). These QTLs were also associated with an increased percentage of biological nitrogen derived from atmosphere (%Ndfa) with an additive effect. Such a synergism between root architecture and SNF controlled by QTLs has also been observed in soybean (Yang et al., 2017). The root growth plays an important role in the drought tolerance ability of crop species. Therefore, increased nodulation could be utilized for enhanced drought tolerance in leguminous crops. Two other QTLs associated with nodule number and nodule size have been identified in soybean (Yang et al., 2019). Nodule phenotype and number linked to these QTLs were genotype dependent. A symbiotic interaction between soybean and *Bradyrhizobium* strains was influenced by the QTLs involved in the secretion of genistein by the host roots (Ramongolalaina et al., 2018). The mapped QTLs showed a phenotypic variation under many environmental conditions related to the N level in soil, greenhouse vs. field studies, and the genotypes employed. Efforts to saturate the genetic map may allow the identification of other QTLs controlling SNF which are less affected by the environmental conditions. The usefulness of a QTL depends on the size and stability of the effect across environments and genetic backgrounds, as well as the phenotyping effort required to assess the trait directly (Scott et al., 2020). Despite several advantages of QTL mapping in biparental populations, this approach suffers from low genetic diversity and fewer recombinant events. The drawback is addressed through multi-parent populations, which can generate a mosaic of alleles and, hence, a greater potential of precise and dense mapping in contrast to biparental populations. Two popular approaches, namely, the nested association mapping (NAM) and multi-parent advanced generation inter-cross (MAGIC) populations, are in vogue (reviewed by Scott et al., 2020). Many of the agronomic traits, though not directly linked to symbiosis, have been mapped in common bean, faba bean, and cowpea through MAGIC populations (Diaz et al., 2020; Scott et al., 2020). MAGIC germplasm is a valuable resource for SNF-QTL mapping in legumes.

Developments in genome analysis have led to new approaches to study phenotypic variations linked to the genetic makeup. Genome-wide association studies (GWAS), which involve hundreds and thousands of single-nucleotide polymorphisms (SNPs) in DNA, have emerged as a powerful tool in associating genetic changes with quantitative trait variation in plants and animals (Zhu et al., 2018). GWAS allows detection of causative alleles or loci which sometimes are not possible through QTL mapping. The use of genome sequencing has led to a significant progress in trait mapping in chickpea, pigeon pea, and groundnut (reviewed by Varshney et al., 2019). In many such studies, yield was the primary focus followed by disease resistance. Although higher SNF efficiency correlated with higher yields, there was little or no emphasis on traits linked to improvement in SNF in these studies. Nonetheless, candidate genes for SNF and significant SNPs have been identified by GWAS on Pv03, Pv07, and Pv09 chromosomes of common bean (Kamfwa et al., 2015, 2019). Two of the candidate genes code for leucine-rich repeat receptor-like protein kinases that play a key role in signal transduction during nodulation (Sanchez-Lopez et al., 2011).

The cell wall β-expansins encoded by *GmINS1* and *GmEXPB2* promote nodule development in soybean (Li et al., 2015, 2018). SNF in *Lotus japonicus* required the presence of sulfate transporters (SST1) and certain membrane proteins (Krusell et al., 2005; Kumagai et al., 2007). Sequencing of red clover phenotypes differentiating in SNF resulted in the identification of two genes, ethylene response factor required for nodule differentiation and molybdate transporter 1 (Trněný et al., 2019). A study in *Medicago truncatula* identified several nodulation-related genes, *SERK2, MtnodGRP3, MtMMPL1, NFP, CaML3*, and *MtnodGRP3A*, which contributed to variations in the legume–rhizobia symbiosis and nodulation (Stanton-Geddes et al., 2013). Similarly, GWAS enabled the identification of several SNPs associated with Ndfa under different environments in soybean (Dhanapal et al., 2015). This approach identified 11 SNF-related characteristics linked to 20 SNP loci on eight chromosomes in soybean (Huo et al., 2019). Among these, three SNP loci located on chromosome Gm17 were associated with the shoot N concentration while two SNP loci in the adjacent region were linked to high nodule numbers and nodule fresh and dry weights. Functional analysis of the candidate genes involved in legume–rhizobia symbiosis has been studied by combining GWAS with newly developed gene disruption technologies such as CRISPR (Curtin et al., 2017). The transcriptomic analysis provides another high-throughput tool to relate to a gene function. Using this technique, involvement of the *GmPAP12* gene in nodule development was demonstrated (Wang et al., 2020a).

The rapid gene sequencing and genome editing tools have accelerated the progress in functional genomics. These, along with mutagenesis techniques, have identified about 200 genes involved in various stages of the symbiotic process in both model and commercial legume crops (reviewed by Roy et al., 2020). The repertoire of genes provides a valuable resource for the breeders who can employ them as markers in MAS, and these could be edited for an efficient nodulation and nitrogen fixation. Even though GWAS serves as a powerful tool to assess the association of genotypic changes with the phenotypes, it presents limitations when the variants either are at low frequency or have a small effect size (Korte and Farlow, 2013). For complex traits such as nodulation, the variants sometimes explain only a small proportion of the heritability. Given that the environment plays a pivotal role in determining a phenotype, the missing heritability could be due to gene–environment interactions. This aspect of heritability has been addressed through gene–environment-wide association studies (Thomas, 2010). The genetic diversity or presence of SNPs in host plant QTLs or genes involved in SNF is an important determinant of the efficiency and capacity of SNF. Equally important is the genetics of participating nitrogen-fixing bacteria, especially the genetic linkages with phenotypic variations. The genomic studies have significantly broadened our knowledge in this area (Sablok et al., 2017; Aguilar et al., 2018; Sánchez-Cañizares et al., 2018).

Comparative genomic studies on a large number of rhizobial genomes have been conducted to identify the putative proteins involved in symbiosis (Queiroux et al., 2012; Seshadri et al., 2015). Some of these proteins are highly expressed in nodules than in a free-living bacterium. Using a GWAS approach in *Ensifer meliloti* strains, the phenotypic variations that most strongly associated with symbiosis phenotypes were identified and linked to the genes involved in nitrogen fixation or nodulation (Epstein et al., 2018). A QTL mapping study in soybean recombinant inbred lines (RILs) involving a symbiosis with *Sinorhizobium fredii* HH103ΩrhcJ mutant, which resulted in a reduced nodule number, identified three host genes that might be participating in the reduction process (Zhu et al., 2019). Another protein, NopD, which is one of the type III effectors of *S. fredii*, influences the expression of two genes associated with two QTLs (Wang et al., 2020b). These interactions resulted in nodule phenotypic differences in soybean RILs. The identification of QTLs, genes associated with them, and SNPs are promising not only for improving our understanding of host–rhizobia symbiotic interaction but also for providing vital tools for molecular breeding of leguminous crops for enhanced SNF efficiency.

### Rhizobial–Host Interaction And SNF Efficiency

**FIGURE 2:**
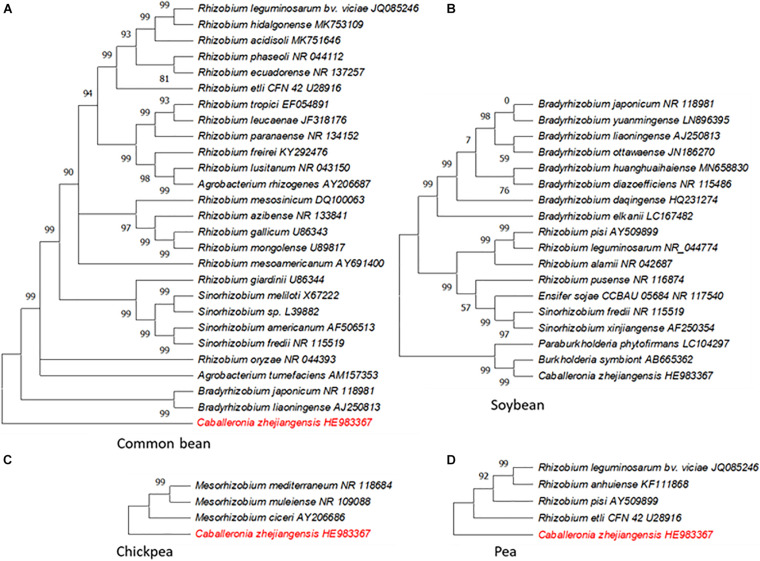
Genetic diversity of rhizobia associated with different crops for biological nitrogen fixation. **(A)** Common bean. **(B)** Soybean. **(C)** Chickpea. **(D)** Pea. The phylogenetic trees of 16S ribosomal RNA of the rhizobial species known to nodulate the indicated crop species were constructed in MEGA X (Kumar et al., 2018) using the neighbor-joining method. *Caballeronia zhejiangensis* HE983367 displayed in red font in **A, C**, and **D** was used as a reference sequence in the analysis; this rhizobial species does not forge symbiosis with these crops.

Symbiosis is a highly complex and structured process where both the host and rhizobial strain contribute in establishing the latter as a microsymbiont in root nodules (reviewed by Roy et al., 2020). To enhance the role of legume crops in agriculture sustainability, ideally there is a need for proficient symbiotic interaction(s) between the host and rhizobia for a maximum N output. Rhizobia display host preferences with varying degrees of specificity while the hosts also favor some strains over others (Andrews and Andrews, 2017). The host range of rhizobia is largely determined by nodulating genes with their counterparts in host plants (Perret et al., 2000). Neither rhizobial strains that can nodulate all leguminous plants nor any legume plant known to develop symbiosis with all strains of rhizobia have been identified. This suggested that the specificity is controlled through a regulatory mechanism present in both the host and rhizobial strain. Some legumes display a rigid regime of symbiotic bacteria preference while others tend to be flexible. Common bean (*Phaseolus vulgaris*) and soybean (*Glycine max*), for example, can be nodulated by a large number of taxonomically different rhizobia including members of genus *Burkholderia*, while in pea (*Pisum sativum*) and chickpea (*Cicer arietinum*) the symbiosis is restricted to a fairly narrow range of rhizobial strains (Andrews and Andrews, 2017; Muñoz-Azcarate et al., 2017; Ramírez et al., 2019; Figure 2).

Both pea and chickpea, and many other agriculturally important legumes, belong to an inverted repeat lacking clade (IRLC). This clade is marked by a loss of inverted repeats in the plastid genome which results in its size variation (Wojciechowski et al., 2004). All the members of IRLC have indeterminate nodules and are known to possess a high degree of host specificity. IRLC members are commonly nodulated by *Ensifer*, *Mesorhizobium*, and *Rhizobium* spp. but rarely with the members of *Bradyrhizobium*, *Neorhizobium*, and *Phyllobacterium* genera. Further, nearly all *Medicago*, *Melilotus*, and *Trigonella* spp. are nodulated by either *Ensifer medicae* or *Ensifer meliloti*, while *Trifolium spp*. and members of tribe Fabeae are nodulated by *Rhizobium* spp. (ref. in Sprent et al., 2017). There are no reports of *Burkholderia* or *Cupriavidus* symbionts within the IRLC (Andrews and Andrews, 2017). The specificity of *Rhizobium leguminosarum* bv. *viciae* genotypes with their hosts has been found to be linked to plant-specific SNP patterns observed within the *nod* gene cluster (Jorrin and Imperial, 2015). The rhizobia–host specificity, among many factors, is determined by a set of flavonoids produced by hosts. These chemical signals are perceived by the rhizobial species to produce Nod factors, which are recognized by the host receptors present on the root cell membrane, thereby adding another layer of specificity (Broughton et al., 2000; Wang et al., 2018; Roy et al., 2020). Nod factors are composed of a β-1,4–linked N-acetyl-glucosamine backbone with functional groups attached to the reducing and nonreducing termini (Geurts and Bisseling, 2002). The IRLC legumes, which are known to possess high specificity with symbiotic bacteria, have a stringent requirement of the Nod factors with unsaturated fatty acyl chains (Debellé et al., 2001). A very high specificity within IRLC legumes is associated with a greater N fixation efficiency on the basis of per unit carbon utilized (Oono and Denison, 2010; Kereszt et al., 2011). In contrast, common bean which is a promiscuous host to rhizobial strains is considered as a weak nitrogen fixer compared to other grain legumes (Muñoz-Azcarate et al., 2017).

A better understanding between the host and its rhizobial partner or host–rhizobia specificity provides a gateway for an efficient symbiosis and nitrogen output. The genetic diversity of rhizobia in the soil offers opportunities for a range of mutualistic interactions. The environmental factors, soil management practices, and cultivation history significantly influence the genetic diversity (Kaschuk et al., 2006; Mothapo et al., 2013; Yan et al., 2014). Almost each member of the rhizobial community tends to exploit its genetics to forge a symbiotic relationship with the available hosts. Because the competitiveness and N_2_ fixing efficiency are independent traits, there is a high probability that more competitive rhizobia with poor-performing capability will colonize the host along with others, and their proportion in the colonized population will be an important determinant of SNF efficiency (Burghardt et al., 2018). As a counter mechanism, the host displays an ability to sanction the nonperforming symbionts by cutting off the nutrient supply (Westhoek et al., 2017), but what level of nonperformance triggers this mechanism is not understood. Such an action is expected to have a cost, both in terms of resources and time. Therefore, it may not be advantageous for the host to sanction every bacterium which is not the best performing. This may explain in part why a very high specificity in IRLC legumes is associated with more efficient N_2_ fixation compared to promiscuous legumes. Further understanding on specificity and competitiveness should provide cues for developing elite strains with optimized symbiotic output.

A breakthrough research has enabled the simultaneous measurement of rhizobial competitiveness and N_2_ fixation in nodules (Mendoza-Suárez et al., 2020). The studies indicate that introduction of modified strains for improved SNF efficiency and other symbiotic characteristics face a tough competition for their performance from the native strains (Goyal et al., 2021). An indigenous rhizobia can dilute or even abolish the positive effect of an inoculant (Dwivedi et al., 2015). A study with six different legume species inoculated with superior N_2_-fixing strains proved ineffective over native soil rhizobia to enhance N_2_ fixation (Singleton and Tavares, 1986). Similarly, the commercial inoculants did not provide a significant benefit in nodulation and crop yield over native strains (Nyaga and Njeru, 2020). Exceptionally, the native strains outperform commercial strains for N_2_-fixation effectiveness in different legume crops (Ouma et al., 2016; Koskey et al., 2017; ref. in Mendoza-Suárez et al., 2020). The nonnative inoculants face competition from the native strains and may be unable to maintain their superiority. Rhizobial populations vary from highly beneficial to ineffective in natural and agricultural soils, and the community-level effects seem to favor the persistence of ineffective rhizobia (Pahua et al., 2018). It is known that the microbial communities cause an either favorable or unfavorable environment for specific rhizobia in the rhizosphere, thus creating unequal opportunities for the strains (Han et al., 2020). These studies underscore the importance of native strains for developing more competitive or better-performing inoculants since nodule occupancy by the natives tends to be significantly higher (Irisarri et al., 2019). In view of these observations, an SNF improvement program faces challenges in developing and deploying superior inoculants in crop production systems each having a unique ecosystem. Nevertheless, the drag due to a mix of poor-performing strains presents an opportunity to maximize the nodule occupancy with efficient strains for an efficient SNF.

## Environmental Factors Affecting SNF

**FIGURE 3:**
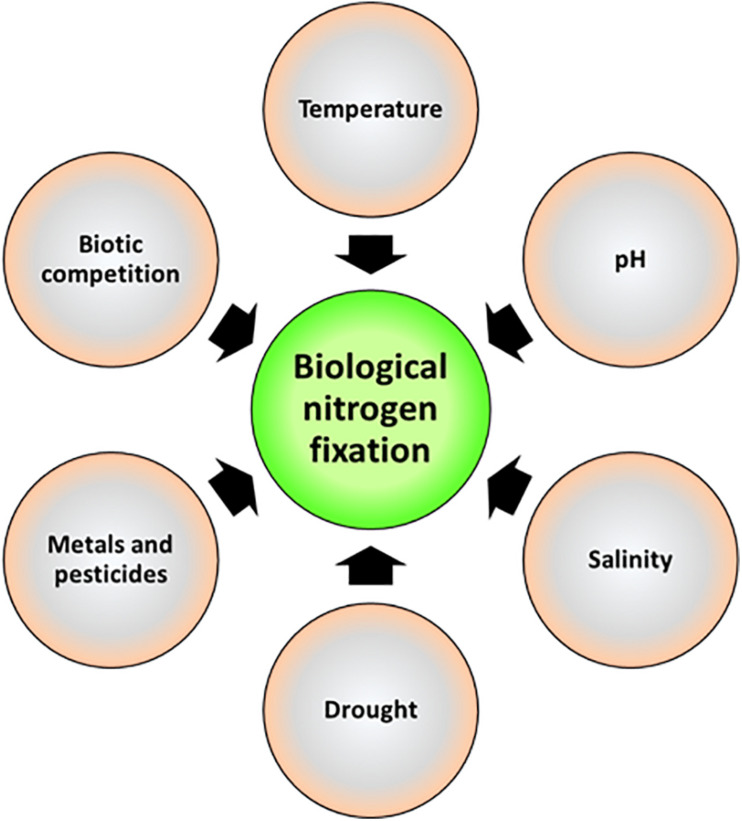
Prominent factors affecting the symbiosis and efficiency of biological nitrogen fixation.

Environmental factors have profound effects on rhizobial diversity, which is determined by the adaptability of microbial species to the prevailing conditions (Voung et al., 2017; Koskey et al., 2018). Environmental stress, which affects both the host and rhizobial response, leads to reduction in SNF efficiency (Laranjo and Oliveira, 2011). The magnitude of reduction depends on how the bacteria live, thrive, and survive under those environments given that the dynamics of stress changes rapidly under combined extremes of temperature, salinity, drought, soil pH, pesticides, or nutrient deficiency (Hungria and Vargas, 2000). The prominent factors that influence the symbiotic process are discussed below (Figure 3). Genetic improvements of strains for better adaptation to a specific stress have been recently reviewed (Goyal et al., 2021).

### Temperature

Temperature significantly affects the host–rhizobia interactions and determines geographical distribution of strains and their ability to nodulate (Hungria and Vargas, 2000). Each legume–rhizobia combination tends to display specificities for an optimal performance (Igiehon et al., 2019). For many rhizobia, the ideal temperature range is 25–30°C, but the strains are known to fix nitrogen between 30 and 42°C in arid and semiarid conditions (Dwivedi et al., 2015). Temperature in these regions, especially those with sandy soils, can touch 60°C. Bacterial survival is key for a symbiotic process when favorable conditions return. Strain adaptation to high temperatures has been observed for certain rhizobial species, although these are usually associated with reduction in nodule weight, number, and nitrogenase activity leading to decreased N fixation (Baldani and Weaver, 1992; Rodrigues et al., 2006; Ormeño-Orrillo et al., 2016). Downregulation of the *nodC* gene has been observed at higher than optimal temperatures (Shiro et al., 2016), whereas a complete abolition of nodulating ability could be due to curing of the plasmids contributing to N fixation (Hungria and Franco, 1993). Further, significant variations have been observed among different host–rhizobial interactions at low temperature. In pigeon pea (*Cajanus cajan*) and cowpea (*Vigna unguiculata*) *Bradyrhizobium* symbioses, the N fixation ceases below 21°C (Marsh et al., 2006), while in soybean the complete inhibition of nodulation occurs below 10°C (Matthews and Hayes, 1982). However, some strains display greater resiliency by fixing N even at 4°C (Yuan et al., 2020). Low temperature reduces secretion of the flavonoids involved in signaling of nodule formation, which also could contribute to poor N fixation by the rhizobia (reviewed by Wang et al., 2018). A substantial decrease in the level of genistein was noted below 17.5°C (Zhang and Smith, 1996). Application of genistein and daidzein stimulated the nodulation and nitrogen fixation under a low-temperature regime (Broughton et al., 2003). Along with reduction in flavonoids at low temperatures, a decline in the excretion of nod factors was also observed in *Rhizobium leguminosarum* bv. *trifolii* (McKay and Djordjevic, 1993) and *Bradyrhizobium* (Zhang et al., 2002). Overall, these studies indicate that stress-induced impairment in the signaling mechanism leads to slower or inhibition of nodule formation (Lira et al., 2015). The molecular changes in response to temperature stress are discussed in detail elsewhere (Alexandre and Oliveira, 2013; da-Silva et al., 2017).

### pH

Certain agricultural practices and industrial pollution tend to make the soil acidic with a negative impact on crop productivity. An acidic soil with pH below 5.5 is considered poor in quality, which exacerbates the metal ion toxicity. These factors affect the growth and symbiotic characteristics of rhizobia, thereby influencing their distribution, survival, and nodulating ability (Ferguson et al., 2013; Lira et al., 2015). The sensitive strains in acidic soils are compromised for N fixation, which can significantly affect the legume production unless fertilized (Hungria and Vargas, 2000). A reduced secretion of flavonoids and Nod factors appears to be responsible in part for the lower number of nodules under acidic conditions (McKay and Djordjevic, 1993). Considerable structural change in the profile of Nod factors occurs when the pH is lowered from neutral to acidic (Morón et al., 2005). In general, low pH does not favor the growth of rhizobia, but there is a wide variation across different strains in response to pH. Some strains of *Mesorhizobium ciceri* found to be moderately acidophilic prefer a pH range of 5–7 for their growth (Laranjo and Oliveira, 2011). Another study on *Rhizobium tropici*, *Rhizobium cellulosilyticum, Rhizobium taibaishanense*, and *Sinorhizobium meliloti* found that certain strains were able to grow at pH 4, indicating their adaptability to environmental fluctuations (Igiehon et al., 2019). Rhizobia employ mechanisms related to decreased membrane permeability, internal buffering, and prevention of metal ion toxicity to maintain ideal intracellular pH (Ormeño-Orrillo et al., 2016). The acid-tolerance response involves multiple genes spanning intracellular signaling to metabolic adjustments (Draghi et al., 2016), which presents challenges in strain improvement for acid tolerance.

### Salinity

It is estimated that 40% of the available land surface area is affected by salinity and desiccation, thereby severely limiting the productivity potential of crops. Both bacteria and plants experience adverse effects of salinity, reducing thereby the availability of water and causing water deficiency or desiccation. Salinity stress has significant consequences on the survival, colonization, and nodule activity of rhizobia (Vriezen et al., 2007; Brígido et al., 2012). A differential response of rhizobial strains to salt tolerance is an indication of their evolutionary fitness to the environment (Burghardt, 2020). *Bradyrhizobium* strains are among the most sensitive to a high-salt environment followed by *Mesorhizobium*, while *Rhizobium* and *Sinorhizobium* are relatively more tolerant (Laranjo and Oliveira, 2011; Brígido et al., 2012). The increased concentration of low molecular weight solutes is one of the several mechanisms observed in salt-tolerant strains (Dong et al., 2017; Furlan et al., 2017). To increase the biosynthesis of trehalose, the *M. ciceri* strain was modified to improve tolerance to salt as well as symbiosis with the host (Moussaid et al., 2015). Similarly, a salt-tolerant *Bradyrhizobium* strain accumulated more trehalose, proline, and betaine, among other compounds, which led to higher number of nodules, plant dry weight, and nitrogen level (Dong et al., 2017). Salt stress may diminish the capacity of bean root exudates to induce *nod* genes for producing lipochitooligosaccharides (Nod factors), while it did not change the pattern of flavonoids (Dardanelli et al., 2012). In another study with a salt-tolerant strain *R. tropici* CIAT 899, induction of *nod* genes and production of Nod factors were observed even under high concentrations of salt (Guasch-Vidal et al., 2013). An RNA-seq analysis of *R. tropici* CIAT 899 under salt stress revealed that nodulation genes were expressed in the absence of flavonoids (Pérez-Montaño et al., 2016). Moreover, the same set of symbiotic genes was upregulated in the presence of both a Nod-factor inducer apigenin and salt. These studies suggest the presence of different dynamics of flavonoid-nod factor signaling in salt-sensitive vs. salt-tolerant strains.

### Drought

Lack of moisture in soil for extended time periods causes drought and affects plant growth and development as well as microbial activities. Under drought conditions, a decrease in nodule number, biomass, and nodule-specific activity resulted in a substantial reduction in biologically derived nitrogen acquisition (Serraj et al., 1999; Marquez-Garcia et al., 2015). A precise mechanism of reduction in nitrogenase activity was not deciphered, but a limiting supply of photosynthates for the nodule and loss of leghemoglobin were considered as playing a role (Serraj et al., 1999). Drought stress altered the morphology of legume roots and accelerated the senescence of nodules in soybean (Kunert et al., 2016). Exogenous application of a Nod factor under drought conditions led to a higher cytokinin level in plant organs and coincided with increased nodule number and nodule-specific activity (Prudent et al., 2016). Other studies have indicated that drought induces similar changes in both determinate- and indeterminate-type nodules (Gil-Quintana et al., 2015). The differences in gene expression were attributed to different nodule types (Sańko-Sawczenko et al., 2019). Dehydration of the cell membrane causes damage to the lipid bilayer, and this can jeopardize the bacterial survival during drought (Bushby and Marshall, 1977). A slow rate of desiccation that led to accumulation of osmoprotectants was found to improve rhizobia survival for a longer time period (Antheunisse et al., 1981). A correlative study demonstrated that the nodules of drought-tolerant soybean cultivar accumulated higher levels of trehalose, proline, and betaine compared to the susceptible cultivar (Furlan et al., 2017). Likewise, a double mutation in *ostA* and *treY* genes impaired the accumulation of trehalose and increased the vulnerability of a *R. leguminosarum* bv. *trifolii* strain to desiccation (McIntyre et al., 2007). These studies highlight the importance of osmoprotectants in drought tolerance. Furthermore, extreme water status, i.e., excess of water in waterlogged conditions, also severely impairs nodule activity and N fixation (see a review by Pucciariello et al., 2019). A study on the role of nitric oxide homeostasis revealed that a mutation in a single domain of hemoglobin in *Bradyrhizobium diazoefficiens* conferred protection to symbiotic nitrogen fixation during flooding in soybean plants (Salas et al., 2020).

### Metals and Pesticides

The Industrial Revolution significantly contributed to heavy metal contamination of the soil, affecting microbial activities and influencing their diversity and population. The most common metals contaminating the soil are lead (Pb), cadmium (Cd), arsenic (As), zinc (Zn), chromium (Cr), copper (Cu), mercury (Hg), and nickel (Ni) (Li et al., 2019). Although Cu, Ni, Co, and Zn are required as micronutrients, at high concentrations they are toxic to plants and microorganisms. Similarly, aluminum (Al), which is the most abundant metal in Earth’s crust, causes toxicity under acidic conditions (Bojórquez-Quintal et al., 2017). Below pH 5.0, Al exists in its trivalent form (Al^3+^), which exerts its toxic effect on many aspects of the rhizobia/legume symbiosis, consequently impairing the nitrogen fixation process (reviewed by Jaiswal et al., 2018). An excessive level of Al under low pH conditions reduced nitrogenase activity by as much as 50% in addition to biochemical changes in the nodules during the *Phaseolus vulgaris*–*R. tropici* CIAT899 symbiosis (Mendoza-Soto et al., 2015). The prominent mechanisms of Al toxicity mitigation in rhizobia include induction of efflux pumps, reduction in Al uptake through synthesis of siderophores, production of exopolysaccharides, and synthesis of citric acid (Artigas Ramírez et al., 2018; Jaiswal et al., 2018). Heavy metal contamination of the soil has negative effects on plant growth and development as well as symbiotic activity (DalCorso, 2012). In a peanut–*Bradyrhizobium* sp symbiotic interaction, 10 μM Cd reduced both the nodule number and the N content (Bianucci et al., 2013). Similarly, the contamination of Cu in soil reduced the nodule number in common bean, causing a shift in rhizobial communities that were tolerant to Cu (Laguerre et al., 2006). Chromium, which naturally occurs in its two predominant ionic states, Cr^+6^ and Cr^+3^, causes a very strong oxidative stress resulting in adverse effects on symbiotic interactions (Stambulska et al., 2018). Induction of antioxidant defense is one of the mechanisms *Rhizobium* deploys against metal-induced oxidative stress. Metal-tolerant rhizobia cause an efflux of metals. An interference in a *dmeF* gene that plays a role in metal efflux led to the tolerant strains of *R. leguminosarum* bv. *viciae* to become susceptible to Ni and Co toxicity (Rubio-Sanz et al., 2018). The other strategies include extracellular immobilization, periplasmic allocation, cytoplasmic sequestration, and biotransformation of toxic products to overcome the metal stress (Cardoso et al., 2017, 2018). The mechanisms could be specific to a stress type or may involve a broader approach of reverse phosphorylation for adjustment of several cellular processes (Lipa and Janczarek, 2020).

Pesticides that are predominantly directed at pathogenic microbial pests could also target the beneficial rhizobial species. A few limited studies do not rule out the adverse impact on the symbiotic activity. One of the earlier studies noticed a harmful effect of Captan and Thiram (TMTD) on nodulation in soybean (Chamber and Montes, 1982). Expanding the evaluation to more fungicides such as Apron, Arrest 75W, Crown, and Captan showed a toxic effect on rhizobial survival to varying degrees with negative consequences on nodulation in chickpea (Kyei-Boahen et al., 2001). Crown and Captan reduced nodulation, %Ndfa, and shoot dry matter. Seed application of Thiram and P-Pickel T also had detrimental effects on rhizobial survival and nodulation in pea (Rathjen et al., 2020). In general, many of the organochlorine pesticides, agrichemicals, and environmental contaminants reduce root nodules, lower the rates of nitrogenase activity, and affect plant yield (Fox et al., 2007). In view of the global use of pesticides exceeding 4 million tons per year, more ecological studies are required to assess their impact on agriculture sustainability.

### Biotic Stress

Pests and diseases can potentially cause substantial yield losses, and such harmful effects extend beyond crops to nitrogen-fixing rhizobia. Nematodes, in particular, were found to interfere with the soybean–rhizobia symbiosis and decreased nodule number and size (Hussey and Barker, 1976). In another study, the soybean root infection by *Pratylenchus penetrans* similar to field infestation severely affected nodulation, density of viable bacteroids in nodules, and N fixation by *B. japonicum* (Elhady et al., 2020). A prior infection by soybean mosaic virus also adversely impacted nodulation in soybean (Andreola et al., 2019). Activation of plants’ defense mechanism in *P. vulgaris* in response to a generalist hemibiotrophic plant pathogen, *Colletotrichum gloeosporioides*, was accompanied with a reduced nodule number by the rhizobia (Ballhorn et al., 2014).

## Future Perspectives

In recent years, a significant progress confined to select commercial grain legumes has been made in the identification of QTLs that determine the symbiotic process and N fixation. The adoption of such information in breeding programs depends on recognizing SNF as an important part of agriculture sustainability. Effective and low-cost synthetic versions of N are likely to be a deterring factor till the associated environmental issues become compelling enough to consider environment-friendly options. The strength and effectiveness of identified QTLs would contribute to their incorporation into breeding efforts. More studies on QTLs with expansion and involving other legume crops would strengthen them as candidates of SNF trait.

A varied degree of rhizobial–host preference, which in part is determined by the presence of reciprocal symbiotic genes in host plants, and the environments dictate the symbiotic process and its performance. Consequently, it sets the limit of N amount which can be accrued in a symbiotic relationship. The existing knowledge along with robust soil microbiome profiles can be used to optimize the host–rhizobia interactions, particularly when new legume crops are introduced into the soil and environment. Unraveling the molecular basis of a negative correlation with rhizobial promiscuousness and N fixation efficiency or vice versa should greatly help in improving the symbiotic N outcome. It is also important to note that recombinant DNA technology has contributed in developing the novel strains (germplasm) that are more adaptive to a given environment and have improved efficiency of SNF. The challenges from the native rhizobial microflora notwithstanding, such strains can be integrated for better N output but will require undertaking more ecological studies to allay the fears of genetically modified organisms. In addition, to protect the scope of SNF in N use sustainability, the application of pesticides as an integral agricultural practice needs to be carefully monitored.

## Author Contributions

RG conceptualized and wrote the manuscript. AM and MS contributed to the writing. All authors contributed to the article and approved the submitted version.

## Conflict of Interest

The authors declare that the research was conducted in the absence of any commercial or financial relationships that could be construed as a potential conflict of interest.

## Footnotes

1 https://www.ipcc.ch/sr15/

2 https://www.engineeringtoolbox.com/co2-emission-fuels-d_1085.html

## References

[B1] Aguilar A. Mora Y. Dávalos A. Girard L. Mora J. Peralta H. (2018). Analysis of genome sequence and symbiotic ability of rhizobial strains isolated from seeds of common bean (*Phaseolus vulgaris*). *BMC Genomics* 19:645. 10.1186/s12864-018-5023-0 PMC6117902 30165827

[B2] Alexandre A. Oliveira S. (2013). Response to temperature stress in rhizobia. *Crit. Rev. Microbiol.* 39 219–228. 10.3109/1040841x.2012.702097 22823534

[B3] Ali J. Xu J. Gao Y. Ma X. Meng L. Wang Y. (2017). Harnessing the hidden genetic diversity for improving multiple abiotic stress tolerance in rice (*Oryza sativa* L.). *PLoS One* 12:e0172515. 10.1371/journal.pone.0172515 28278154 PMC5344367

[B4] Andreola S. Rodriguez M. Parola R. Alemano S. Lascano R. (2019). Interactions between soybean, Bradyrhizobium japonicum and Soybean mosaic virus: the effects depend on the interaction sequence. *Funct. Plant Biol.* 46, 1036–1048. 10.1071/FP17361 31575385

[B5] Andrews M. Andrews M. E. (2017). Specificity in legume-rhizobia symbioses. *Intern. J. Mol. Sci.* 18:705. 10.3390/ijms18040705 28346361 PMC5412291

[B6] Antheunisse J. De Bruin-Tol J. W. Van Der Pol-Van Soest M. E. (1981). Survival of microorganisms after drying and storage. *Antonie Van Leeuwenhoek* 47 539–545. 10.1007/bf00443240 7039502

[B7] Artigas Ramírez M. D. Silva J. D. Ohkama-Ohtsu N. Yokoyama T. (2018). In vitro rhizobia response and symbiosis process under aluminum stress. *Can. J. Microbiol.* 64 511–526. 10.1139/cjm-2018-0019 29620430

[B8] Baldani J. I. Weaver R. W. (1992). Survival of clover rhizobia and their plasmid-cured derivatives in soil under heat and drought stress. *Soil Biol. Biochem.* 24 737–742. 10.1016/0038-0717(92)90247-u

[B9] Ballhorn D. J. Younginger B. S. Kautz S. (2014). An aboveground pathogen inhibits belowground rhizobia and arbuscular mycorrhizal fungi in Phaseolus vulgaris. *BMC Plant Biol.* 14:321. 10.1186/s12870-014-0321-4 25429887 PMC4248430

[B10] Beyene Y. Semagn K. Crossa J. Mugo S. Atlin G. N. Meisel B. (2016). Improving maize grain yield under drought stress and non-stress environments in sub-Saharan Africa using marker-assisted recurrent selection. *Crop Sci.* 56 344–353. 10.2135/cropsci2015.02.0135

[B11] Bianucci E. Furlan A. Rivadeneira J. Sobrino-Plata J. Carpena-Ruiz R. O. Tordable Mdel C, et al. (2013). Influence of cadmium on the symbiotic interaction established between peanut (*Arachis hypogaea* L.) and sensitive or tolerant bradyrhizobial strains. *J. Environ. Manage.* 130 126–134. 10.1016/j.jenvman.2013.08.056 24076512

[B12] Bojórquez-Quintal E. Escalante-Magaña C. Echevarría-Machado I. Martínez-Estévez M. (2017). Aluminum, a friend or foe of higher plants in acid soils. *Front. Plant Sci.* 8:1767. 10.3389/fpls.2017.01767 29075280 PMC5643487

[B13] Bourion V. Rizvi S. M. Fournier S. de Larambergue H. Galmiche F. Marget P. (2010). Genetic dissection of nitrogen nutrition in pea through a QTL approach of root, nodule, and shoot variability. *Theor. Appl. Genet.* 121 71–86. 10.1007/s00122-010-1292-y 20180092

[B14] Brígido C. Alexandre A. Oliveira S. (2012). Transcriptional analysis of major chaperone genes in salt-tolerant and salt-sensitive mesorhizobia. *Microbiol. Res.* 167 623–629. 10.1016/j.micres.2012.01.006 22364959

[B15] Broughton W. J. Jabbouri S. Perret X. (2000). Keys to symbiotic harmony. *J. Bacteriol.* 182 5641–5652. 10.1128/jb.182.20.5641-5652.2000 11004160 PMC94683

[B16] Broughton W. J. Zhang F. Perret X. Staehelin C. (2003). Signals exchanged between legumes and Rhizobium: agricultural uses and perspectives. *Plant Soil* 252 129–137. 10.1023/a:1024179717780

[B17] Burghardt L. T. (2020). Evolving together, evolving apart: measuring the fitness of rhizobial bacteria in and out of symbiosis with leguminous plants. *New Phytol.* 228 28–34. 10.1111/nph.16045 31276218

[B18] Burghardt L. T. Epstein B. Guhlin J. Nelson M. S. Taylor M. R. Young N. D. (2018). Select and resequence reveals relative fitness of bacteria in symbiotic and free-living environments. *Proc. Natl. Acad. Sci. U.S.A.* 115 2425–2430. 10.1073/pnas.1714246115 29453274 PMC5877963

[B19] Burias N. Planchon C. (1990). Increasing soybean productivity through selection for nitrogen fixation. *Agron. J.* 82 1031–1034. 10.2134/agronj1990.00021962008200060001x

[B20] Bushby H. V. A. Marshall K. C. (1977). Desiccation-induced damage to the cell envelope of root-nodule bacteria. *Soil Biol. Biochem.* 9 149–152. 10.1016/0038-0717(77)90066-9

[B21] Caffrey K. R. Veal M. W. (2013). Conducting an agricultural life cycle assessment: challenges and perspectives. *Scientific World J.* 2013 472431. 10.1155/2013/472431 24391463 PMC3874300

[B22] Cardoso P. Corticeiro S. Freitas R. Figueira E. (2018). Different efficiencies of the same mechanisms result in distinct Cd tolerance within Rhizobium. *Ecotoxicol. Environ. Safety* 150 260–269. 10.1016/j.ecoenv.2017.12.002 29289861

[B23] Cardoso P. Santos M. Freitas R. Rocha S. M. Figueira E. (2017). Response of Rhizobium to Cd exposure: a volatile perspective. *Environ. Pollu.* 231 802–811. 10.1016/j.envpol.2017.08.067 28865386

[B24] Chamber M. A. Montes F. J. (1982). Effects of some seeds disinfectants and methods of rhizobial inoculation on soybeans (*Glycine max* L. Merrill). *Plant Soil* 66 353–360. 10.1007/bf02183801

[B25] Chaudhary A. Gustafson D. Mathys A. (2018). Multi-indicator sustainability assessment of global food systems. *Nat. Commun.* 9:848. 10.1038/s41467-018-03308-7 29487286 PMC5829192

[B26] Curtin S. J. Tiffin P. Guhlin J. Trujillo D. I. Burghardt L. T. Atkins P. (2017). Validating genome-wide association candidates controlling quantitative variation in nodulation. *Plant Physiol.* 173 921–931. 10.1104/pp.16.01923 28057894 PMC5291020

[B27] DalCorso G. (2012). “Heavy Metal Toxicity in Plants,” in *Plants and Heavy Metals. SpringerBriefs in Molecular Science*, ed. Furini A. (Dordrecht: Springer), 10.1007/978-94-007-4441-7_1

[B28] Dardanelli M. S. De Córdoba F. J. F. Estévez J. Contreras R. Cubo M. T. Rodríguez-Carvajal M. T. (2012). Changes in flavonoids secreted by Phaseolus vulgaris roots in the presence of salt and the plant growth-promoting rhizobacterium *Chryseobacterium balustinum*. *Appl. Soil Ecol.* 57 31–38. 10.1016/j.apsoil.2012.01.005

[B29] da-Silva J. R. Alexandre A. Brígido C. Oliveira S. (2017). Can stress response genes be used to improve the symbiotic performance of rhizobia? *AIMS Microbiol.* 3 365–382. 10.3934/microbiol.2017.3.365 31294167 PMC6604987

[B30] Debellé F. Moulin L. Mangin B. Dénarié J. Boivin C. (2001). Nod genes and nod signals and the evolution of the rhizobium legume symbiosis. *Acta Biochim. Polonica* 48 359–365. 10.18388/abp.2001_3921 11732607

[B31] Dhanapal A. P. Ray J. D. Singh S. K. Hoyos-Villegas V. Smith J. R. Purcell L. C. (2015). Genome-wide association analysis of diverse soybean genotypes reveals novel markers for nitrogen derived from atmosphere (Ndfa), nitrogen concentration ([N]) and C/N ratio. *Plant Genome* 8:elantgenome2014.11.0086. 10.3835/plantgenome2014.11.0086 33228264

[B32] Diaz L. M. Ricaurte J. Cajiao C. Galeano C. H. Rao I. Beebe S. (2017). Phenotypic evaluation and QTL analysis of yield and symbiotic nitrogen fixation in a common bean population grown with two levels of phosphorus supply. *Mol. Breeding.* 37:76. 10.1007/s11032-017-0673-1

[B33] Diaz S. Ariza-Suarez D. Izquierdo P. Lobaton J. D. da la Hoz J. F. Acevedo F. (2020). Genetic mapping for agronomic traits in a MAGIC population of common bean (*Phaseolus vulgaris* L.) under drought conditions. *BMC Genomics* 21:799. 10.1186/s12864-020-07213-6 33198642 PMC7670608

[B34] Dong R. Zhang J. Huan H. Bai C. Chen Z. Liu G. (2017). High salt tolerance of a Bradyrhizobium strain and its promotion of the growth of Stylosanthes guianensis. *Intern. J. Mol. Sci.* 18:1625. 10.3390/ijms18081625 28788047 PMC5578016

[B35] Draghi W. O. Del Papa M. F. Hellweg C. Watt S. A. Watt T. F. Barsch A. (2016). A consolidated analysis of the physiologic and molecular responses induced under acid stress in the legume-symbiont model-soil bacterium *Sinorhizobium meliloti*. *Sci. Rep.* 6:29278. 10.1038/srep29278 27404346 PMC4941405

[B36] Dwivedi S. L. Sahrawat K. L. Upadhyaya H. D. Mengoni A. Galardini M. (2015). “Advances in host plant and rhizobium genomics to enhance symbiotic nitrogen fixation in grain legumes,” in *Advances in Agronomy*, ed. Donald L. (Cambridge, MA: Academic Press Inc).

[B37] Elhady A. Hallmann J. Heuer H. (2020). Symbiosis of soybean with nitrogen fixing bacteria affected by root lesion nematodes in a density-dependent manner. *Sci. Rep.* 10:1619. 10.1038/s41598-020-58546-x 32005934 PMC6994534

[B38] Epstein B. Abou-Shanab R. Shamseldin A. Taylor M. R. Guhlin J. (2018). Genome-wide association analyses in the model Rhizobium Ensifer meliloti. *mSphere* 3:e00386-18. 10.1128/mSphere.00386-18 30355664 PMC6200981

[B39] Fang Y. Zhang X. Xue D. (2019). Genetic analysis and molecular breeding applications of malting quality QTLs in barley. *Front. Genet.* 10:352. 10.3389/fgene.2019.00352 31068969 PMC6491634

[B40] FAO (2017). *World Fertilizer Trends and Outlook to 2020.* Available online at: http://www.fao.org/3/a-i6895e.pdf (accessed November 26, 2020).

[B41] Farid M. (2015). *Symbiotic Nitrogen Fixation in Common Bean. Ph. D, Thises.* Ontario, CA: University of Guelph.

[B42] Ferguson B. J. Lin M.-H. Gresshoff P. M. (2013). Regulation of legume nodulation by acidic growth conditions. *Plant Signal. Behav.* 8 e23426–e23426. 23333963 10.4161/psb.23426 PMC3676511

[B43] Ferguson B. J. Mens C. Hastwell A. H. Zhang M. Su H. Jones C. H. (2019). Legume nodulation: the host controls the party. *Plant Cell Environ.* 42 41–51. 10.1111/pce.13348 29808564

[B44] Fox J. E. Gulledge J. Engelhaupt E. Burow M. E. Mclachlan J. A. (2007). Pesticides reduce symbiotic efficiency of nitrogen-fixing rhizobia and host plants. *Proc. Natl. Acad. Sci. U.S.A.* 104 10282–10287. 10.1073/pnas.0611710104 17548832 PMC1885820

[B45] Furlan A. L. Bianucci E. Castro S. Dietz K. J. (2017). Metabolic features involved in drought stress tolerance mechanisms in peanut nodules and their contribution to biological nitrogen fixation. *Plant Sci.* 263 12–22. 10.1016/j.plantsci.2017.06.009 28818367

[B46] Galloway J. N. Aber J. D. Erisman J. W. Seitzinger S. P. Howarth R. W. Cowling E. B. (2003). The Nitrogen Cascade. *Bio. Sci.* 53 341–356.

[B47] Geurts R. Bisseling T. (2002). Rhizobium Nod factor perception and signaling. *Plant Cell* 14 s239–s249. 10.1105/tpc.002451 12045280 PMC151258

[B48] Gilbert N. (2012). One-third of our greenhouse gas emissions come from agriculture. *Nature* 10.1038/nature.2012.11708

[B49] Gil-Quintana E. Lyon D. Staudinger C. Wienkoop S. González E. M. (2015). Medicago truncatula and *Glycine max*: different drought tolerance and similar local response of the root nodule proteome. *J. Proteome Res.* 14 5240–5251. 10.1021/acs.jproteome.5b00617 26503705 PMC4673605

[B50] Glendining M. J. Dailey A. G. Williams A. G. Evert F. K. V. Goulding K. W. T. Goulding A. P. (2009). Is it possible to increase the sustainability of arable and ruminant agriculture by reducing inputs? *Agric. Syst.* 99 117–125. 10.1016/j.agsy.2008.11.001

[B51] Goyal R. K. Schmidt M. A. Hynes M. F. (2021). Molecular biology in the improvement of biological nitrogen fixation by rhizobia and extending the scope to cereals. *Microorganisms* 9:125. 10.3390/microorganisms9010125 33430332 PMC7825764

[B52] Grunvald A. K. Torres A. R. Passianotto A. L. L. Santos M. A. Jean M. Belzile F. J. (2018). Identification of QTLs associated with biological nitrogen fixation traits in soybean using a genotyping-by-sequencing approach. *Crop Sci.* 58 1–10.

[B53] Guasch-Vidal B. Estévez J. Dardanelli M. S. Soria-Díaz M. E. Fernández De Córdoba F. (2013). High NaCl concentrations induce the nod genes of Rhizobium tropici CIAT899 in the absence of flavonoid inducers. *Mol. Plant Microbe Interact.* 26 451–460. 10.1094/mpmi-09-12-0213-r 23216086

[B54] Han Q. Ma Q. Chen Y. Bing T. Xu L. Bai Y. (2020). Variation in rhizosphere microbial communities and its association with the symbiotic efficiency of rhizobia in soybean. *ISME J* 14 1915–1928. 10.1038/s41396-020-0648-9 32336748 PMC7367843

[B55] Heilig J. A. Beaver J. S. Wright E. M. Song Q. J. Kelly J. D. (2017). QTL analysis of symbiotic nitrogen fixation in a black bean population. *Crop Sci.* 57 118–129. 10.2135/cropsci2016.05.0348

[B56] Hungria M. Franco A. A. (1993). Effects of high temperature on nodulation and nitrogen fixation by *Phaseolus vulgaris* L. *Plant Soil* 149 95–102. 10.1007/bf00010766

[B57] Hungria M. Vargas M. A. T. (2000). Environmental factors affecting N2 fixation in grain legumes in the tropics, with an emphasis on Brazil. *Field Crops Res.* 65 151–164. 10.1016/s0378-4290(99)00084-2

[B58] Huo X. Li X. Du H. Kong Y. Tian R. Li W. (2019). Genetic loci and candidate genes of symbiotic nitrogen fixation–related characteristics revealed by a genome-wide association study in soybean. *Mol. Breeding* 39:127. 10.1007/s11032-019-1022-3

[B59] Hussey R. S. Barker K. R. (1976). Influence of nematodes and light sources on growth and nodulation of soybean. *J. Nematol.* 8:5. PMC2620149 19308196

[B60] Hwang S. Ray J. D. Cregan P. B. King C. A. Davies M. K. Purcell L. C. (2014). Genetics and mapping of quantitative traits for nodule number, weight, and size in soybean (*Glycine max* L.[Merr.]). *Euphytica* 195 419–434. 10.1007/s10681-013-1005-0

[B61] Igiehon N. O. Babalola O. O. Aremu B. R. (2019). Genomic insights into plant growth promoting rhizobia capable of enhancing soybean germination under drought stress. *BMC Microbiol.* 19:159. 10.1186/s12866-019-1536-1 31296165 PMC6624879

[B62] Irisarri P. Cardozo G. Tartaglia C. Reyno R. Gutiérrez P. Lattanzi F. A. (2019). Selection of competitive and efficient rhizobia strains for white clover. *Front. Microbiol.* 2019:768. 10.3389/fmicb.2019.00768 31065250 PMC6489563

[B63] Jaiswal S. K. Naamala J. Dakora F. D. (2018). Nature and mechanisms of aluminium toxicity, tolerance and amelioration in symbiotic legumes and rhizobia. *Biol. Fertility Soils* 54 309–318. 10.1007/s00374-018-1262-0 31258230 PMC6560468

[B64] Jorrin B. Imperial J. (2015). Population genomics analysis of legume host preference for specific rhizobial genotypes in the Rhizobium leguminosarum bv. viciae symbioses. *Mole. Plant Microbe Interact* 28 310–318. 10.1094/MPMI-09-14-0296-FI 25514682

[B65] Kamfwa K. Cichy K. A. Kelly J. D. (2015). Genome-wide association analysis of symbiotic nitrogen fixation in common bean. *Theor. Appl. Genet.* 128 1999–2017. 10.1007/s00122-015-2562-5 26133733

[B66] Kamfwa K. Cichy K. A. Kelly J. D. (2019). Identification of quantitative trait loci for symbiotic nitrogen fixation in common bean. *Theor. Appl. Genet.* 132 1375–1387. 10.1007/s00122-019-03284-6 30671587

[B67] Kaschuk G. Hungria M. Andrade D. S. Campo R. J. (2006). Genetic diversity of rhizobia associated with common bean (*Phaseolus vulgaris* L.) grown under no-tillage and conventional systems in Southern Brazil. *Appl. Soil Ecol.* 32 210–220. 10.1016/j.apsoil.2005.06.008

[B68] Kereszt A. Mergaert P. Kondorosi E. (2011). Bacteroid development in legume nodules: evolution of mutual benefit or of sacrificial victims? *Mole. Plant Microbe Interact.* 24 1300–1309. 10.1094/mpmi-06-11-0152 21995798

[B69] Korte A. Farlow A. (2013). The advantages and limitations of trait analysis with GWAS: a review. *Plant Methods* 9:29. 10.1186/1746-4811-9-29 23876160 PMC3750305

[B70] Koskey G. Mburu S. W. Kimiti J. M. Ombori O. Maingi J. M. Njeru E. M. (2018). Genetic characterization and diversity of Rhizobium isolated from root nodules of mid-altitude climbing bean (*Phaseolus vulgaris* L.) varieties. *Front. Microbiol.* 9:968. 10.3389/fmicb.2018.00968 29867872 PMC5963253

[B71] Koskey G. Mburu S. W. Njeru E. M. Kimiti J. M. Ombori O. Maingi J. M. (2017). Potential of native rhizobia in enhancing nitrogen fixation and yields of climbing beans (*Phaseolus vulgaris* L.) in contrasting environments of eastern Kenya. *Front. Plant Sci.* 2017:8. 10.3389/fpls.2017.00443 PMC5374200 28408912

[B72] Krusell L. Krause K. Ott T. Desbrosses G. Kramer U. Sato S. (2005). The sulfate transporter SST1 is crucial for symbiotic nitrogen fixation in Lotus japonicus root nodules. *Plant Cell* 17 1625–1636. 10.1105/tpc.104.030106 15805486 PMC1091779

[B73] Kumagai H. Hakoyama T. Umehara Y. Sato S. Kaneko T. Tabata S. (2007). A novel ankyrin-repeat membrane protein, IGN1, is required for persistence of nitrogen-fixing symbiosis in root nodules of Lotus japonicus. *Plant Physiol.* 143 1293–1305. 10.1104/pp.106.095356 17277093 PMC1820915

[B74] Kumar J. Gupta D. S. Gupta S. Dubey S. Gupta P. Kumar S. (2017). Quantitative trait loci from identification to exploitation for crop improvement. *Plant Cell Rep.* 36 1187–1213. 10.1007/s00299-017-2127-y 28352970

[B75] Kumar S. Stecher G. Li M. Knyaz C. Tamura K. (2018). MEGA X: Molecular evolutionary genetics analysis across computing platforms. *Mole. Biol. Evol.* 35 1547–1549. 10.1093/molbev/msy096MEGA PMC5967553 29722887

[B76] Kunert K. J. Vorster B. J. Fenta B. A. Kibido T. Dionisio G. Foyer C. H. (2016). Drought stress responses in soybean roots and nodules. *Front. Plant Sci.* 7:1015. 10.3389/fpls.2016.01015 27462339 PMC4941547

[B77] Kyei-Boahen S. Slinkard A. E. Walley F. L. (2001). Rhizobial survival and nodulation of chickpea as influenced by fungicide seed treatment. *Can. J. Microbiol.* 47 585–589. 10.1139/w01-038 11467735

[B78] Ladha J. K. Tirol-Padre A. Reddy C. K. Cassman K. G. Verma S. Powlson D.s. (2016). Global nitrogen budgets in cereals: A 50-year assessment for maize, rice, and wheat production systems. *Sci. Rep.* 6:19355. 10.1038/srep19355 26778035 PMC4726071

[B79] Laguerre G. Courde L. Nouaïm R. Lamy I. Revellin C. Breuil M. C. (2006). Response of rhizobial populations to moderate copper stress applied to an agricultural soil. *Microbiol. Ecol.* 52 426–435. 10.1007/s00248-006-9081-5 16897301

[B80] Laranjo M. Oliveira S. (2011). Tolerance of mesorhizobium type strains to different environmental stresses. Antonie van Leeuwenhoek. *Int. J. Gen. Mol. Microbiol.* 99 651–662. 10.1007/s10482-010-9539-9 21152981

[B81] Leinonen I. Iannetta P. M. P. Rees R. M. Russell W. Watson C. Barnes A. P. (2019). Lysine supply is a critical factor in achieving sustainable global protein economy. *Front. Sustain. Food Syst.* 3:27. 10.3389/fsufs.2019.00027

[B82] Li C. Zhou K. Qin W. Tian C. Qi M. Yan X. (2019). A review on heavy metals contamination in soil: effects, sources, and remediation techniques. *Soil Sediment. Contam. Inter. J.* 28 380–394. 10.1080/15320383.2019.1592108

[B83] Li X. Zhao J. Tan Z. Zeng R. Liao H. (2015). GmEXPB2, a cell wall β-expansin, affects soybean nodulation through modifying root architecture and promoting nodule formation and development. *Plant Physiol.* 169 2640–2653. 26432877 10.1104/pp.15.01029 PMC4677897

[B84] Li X. Zheng J. Yang Y. Liao H. (2018). Increasing nodule size1 expression is required for normal rhizobial symbiosis and nodule development. *Plant Physiol.* 178 1233–1248. 10.1104/pp.18.01018 30266750 PMC6236598

[B85] Lipa P. Janczarek M. (2020). Phosphorylation systems in symbiotic nitrogen-fixing bacteria and their role in bacterial adaptation to various environmental stresses. *PeerJ* 8:e8466. 10.7717/peerj.8466 32095335 PMC7020829

[B86] Lira M. A. Jr. Nascimento L. R. S. Fracetto G. G. M. (2015). Legume-rhizobia signal exchange: promiscuity and environmental effects. *Front. Microbiol.* 6:945. 10.3389/fmicb.2015.00945 26441880 PMC4561803

[B87] MacWilliam S. Wismer M. Kulshreshtha S. (2014). Life cycle and economic assessment of western canadian pulse systems: the inclusion of pulses in crop rotations. *Agric. Syst.* 123 43–53. 10.1016/j.agsy.2013.08.009

[B88] Marquez-Garcia B. Shaw D. Cooper J. W. Karpinska B. Quain M. D. Makgopa E. M. (2015). Redox markers for drought-induced nodule senescence, a process occurring after drought-induced senescence of the lowest leaves in soybean (*Glycine max*). *Ann. Bot.* 116 497–510. 10.1093/aob/mcv030 25851140 PMC4577989

[B89] Marsh L. E. Baptiste R. Marsh D. B. Trinklein D. Kremer R. J. (2006). Temperature effects on *Bradyrhizobium* spp. growth and symbiotic effectiveness with pigeonpea and cowpea. *J. Plant Nutr.* 29 331–346. 10.1080/01904160500476921

[B90] Matthews D. J. Hayes P. (1982). Effect of root zone temperature on early growth, nodulation and nitrogen fixation in soya beans. *J. Agric. Sci.* 98 371–376. 10.1017/s0021859600041915

[B91] McIntyre H. J. Davies H. Hore T. A. Miller S. H. Dufour J. P. Ronson C. W. (2007). Trehalose biosynthesis in *Rhizobium leguminosarum* bv. trifolii and its role in desiccation tolerance. *Appl. Environ. Microbiol.* 73 3984–3992. 10.1128/aem.00412-07 17449695 PMC1932737

[B92] McKay I. A. Djordjevic M. A. (1993). Production and excretion of nod metabolites by *Rhizobium leguminosarum* bv. trifolii are disrupted by the same environmental factors that reduce nodulation in the field. *Appl. Environ. Microbiol.* 59 3385–3392. 10.1128/aem.59.10.3385-3392.1993 16349071 PMC182463

[B93] Mendoza-Soto A. B. Naya L. Leija A. Hernández G. (2015). Responses of symbiotic nitrogen-fixing common bean to aluminum toxicity and delineation of nodule responsive microRNAs. *Front. Plant Sci.* 6:587. 10.3389/fpls.2015.00587 26284103 PMC4519678

[B94] Mendoza-Suárez M. A. Geddes B. A. Sánchez-Cañizares C. Ramírez-González R. H. Kirchhelle C. Jorrin B. (2020). Optimizing *Rhizobium-legume* symbioses by simultaneous measurement of rhizobial competitiveness and N2 fixation in nodules. *Proc. Natl. Acad. Sci. U.S.A.* 117 9822–9831. 10.1073/pnas.1921225117 32317381 PMC7211974

[B95] Morón B. Soria-Díaz M. E. Ault J. Verroios G. Noreen S. Rodríguez-Navarro D. N. (2005). Low pH changes the profile of nodulation factors produced by *Rhizobium tropici* CIAT899. *Chem. Biol.* 12 1029–1040. 10.1016/j.chembiol.2005.06.014 16183027

[B96] Mothapo N. V. Grossman J. M. Maul J. E. Shi W. Isleib T. (2013). Genetic diversity of resident soil rhizobia isolated from nodules of distinct hairy vetch (*Vicia villosa* Roth) genotypes. *Appl. Soil Ecol.* 64 201–213. 10.1016/j.apsoil.2012.12.010

[B97] Moussaid S. Domínguez-Ferreras A. Muñoz S. Aurag J. Berraho E. B. Sanjuán J. (2015). Increased trehalose biosynthesis improves *Mesorhizobium ciceri* growth and symbiosis establishment in saline conditions. *Symbiosis* 67 103–111. 10.1007/s13199-015-0338-y

[B98] Muñoz N. Qi X. Li M.-W. Xie M. Gao Y. Cheung M. Y. (2016). Improvement in nitrogen fixation capacity could be part of the domestication process in soybean. *Heredity* 117 84–93. 10.1038/hdy.2016.27 27118154 PMC4949726

[B99] Muñoz-Azcarate O. González A. M. Santalla M. (2017). Natural rhizobial diversity helps to reveal genes and QTLs associated with biological nitrogen fixation in common bean. *AIMS Microbiol.* 3 435–466. 10.3934/microbiol.2017.3.435 31294170 PMC6604995

[B100] Nicolás M. F. Hungria M. Arias C. A. A. (2006). Identification of quantitative trait loci controlling nodulation and shoot mass in progenies from two Brazilian soybean cultivars. *Field Crops Res.* 95 355–366. 10.1016/j.fcr.2005.04.012

[B101] Nodari R. O. Tsai S. M. Gusmán P. Gilbertson R. L. Gepts P. (1993). Towards an integrated linkage map of common bean III. Mapping genetic factors controlling host-bacteria interactions. *Genetics* 134 341–350. 10.1093/genetics/134.1.341 8514141 PMC1205436

[B102] Nyaga J. W. Njeru E. M. (2020). Potential of native rhizobia to improve cowpea growth and production in semiarid regions of Kenya. *Front. Agron.* 2:606293. 10.3389/fagro.2020.606293

[B103] Ohlson E. W. Seido S. L. Mohammed S. Santos C. A. F. Timko M. P. (2018). QTL mapping of ineffective nodulation and nitrogen utilization-related traits in the IC-1 mutant of cowpea. *Crop Sci.* 58 264–272. 10.2135/cropsci2017.07.0439

[B104] Oono R. Denison R. F. (2010). Comparing symbiotic efficiency between swollen versus nonswollen rhizobial bacteroids. *Plant Physiol.* 154 1541–1548. 10.1104/pp.110.163436 20837702 PMC2971627

[B105] Ormeño-Orrillo E. Gomes D. F. Del Cerro P. Vasconcelos A. T. R. Canchaya C. Almeida L. G. (2016). Genome of *Rhizobium leucaenae* strains CFN 299T and CPAO 29.8: Searching for genes related to a successful symbiotic performance under stressful conditions. *BMC Genomics* 17:534. 10.1186/s12864-016-2859-z 27485828 PMC4971678

[B106] Ouma E. W. Asango A. M. Maingi J. Njeru E. M. (2016). Elucidating the potential of native rhizobial isolates to improve biological nitrogen fixation and growth of common bean and soybean in smallholder farming systems of Kenya. *Int. J. Agron.* 2016:4569241.

[B107] Pahua V. J. Stokes P. J. N. Hollowell A. C. Regus J. U. Gano-Cohen K. A. Wendlandt C. E. (2018). Fitness variation among host species and the paradox of ineffective rhizobia. *J. Evol. Biol.* 31 599–610. 10.1111/jeb.13249 29418031

[B108] Pazdernik D. L. Graham P. H. Vance C. P. Orf J. H. (1996). Host genetic variation in the early nodulation and dinitrogen fixation of soybean. *Crop Sci.* 36 1102–1107. 10.2135/cropsci1996.0011183x003600050005x

[B109] Peoples M. B. Brockwell J. Herridge D. F. Rochester I. J. Alves B. J. R. Urquiaga S. (2009). The contributions of nitrogen-fixing crop legumes to the productivity of agricultural systems. *Symbiosis* 48 1–17. 10.1007/bf03179980

[B110] Pereira P. A. A. Miranda B. D. Attewell K. A. Kmiecik K. A. Bliss F. A. (1993). Selection for increased nodule number in common bean (*Phaseolus vulgaris* L.). *Plant Soil* 148 203–209. 10.1007/BF00012858

[B111] Pérez-Montaño F. Del Cerro P. Jiménez-Guerrero I. López-Baena F. J. Cubo M. T. (2016). RNA-seq analysis of the *Rhizobium tropici* CIAT 899 transcriptome shows similarities in the activation patterns of symbiotic genes in the presence of apigenin and salt. *BMC Genomics* 17:198. 10.1186/s12864-016-2543-3 26951045 PMC4782375

[B112] Perret X. Staehelin C. Broughton W. J. (2000). Molecular basis of symbiotic promiscuity. *Microbiol. Mol. Biol. Rev.* 64 180–201. 10.1128/mmbr.64.1.180-201.2000 10704479 PMC98991

[B113] Prudent M. Salon C. Smith D. L. Emery R. J. N. (2016). Nod factor supply under water stress conditions modulates cytokinin biosynthesis and enhances nodule formation and N nutrition in soybean. *Plant Signal. Behav.* 11:e1212799. 10.1080/15592324.2016.1212799 27454159 PMC5058462

[B114] Pucciariello C. Boscari A. Tagliani A. Brouquisse R. Perata P. (2019). Exploring legume-rhizobia symbiotic models for waterlogging tolerance. *Front. Plant Sci.* 10:578. 10.3389/fpls.2019.00578 31156662 PMC6530402

[B115] Queiroux C. Washburn B. K. Davis O. M. Stewart J. Brewer T. E. Lyons M. R. (2012). A comparative genomics screen identifies a Sinorhizobium meliloti 1021 sodM-like gene strongly expressed within host plant nodules. *BMC Microbiol.* 12:74. 10.1186/1471-2180-12-74 22587634 PMC3462710

[B116] Ramaekers L. Galeano C. H. Garzón N. Vanderleyden J. Blair M. W. (2013). Identifying quantitative trait loci for symbiotic nitrogen fixation capacity and related traits in common bean. *Mol. Breeding* 31 163–180. 10.1007/s11032-012-9780-1

[B117] Ramírez M. D. A. España M. Aguirre C. Kojima K. Ohkama-Ohtsu N. Sekimoto H. (2019). Burkholderia and Paraburkholderia are predominant soybean rhizobial genera in Venezuelan soils in different climatic and topographical regions. *Microbes Environ.* 34 43–58. 10.1264/jsme2.me18076 30773514 PMC6440732

[B118] Ramongolalaina C. Teraishi M. Okumoto Y. (2018). QTLs underlying the genetic interrelationship between efficient compatibility of Bradyrhizobium strains with soybean and genistein secretion by soybean roots. *PLoS One* 13:e0194671. 10.1371/journal.pone.0194671 29617389 PMC5884529

[B119] Rathjen J. R. Ryder M. H. Riley I. T. Lai T. V. Denton M. D. (2020). Impact of seed-applied pesticides on rhizobial survival and legume nodulation. *J. Appl. Microbiol.* 129 389–399. 10.1093/femsle/fnaa084 32011051

[B120] Raun W. R. Johnson G. V. (1999). Improving nitrogen use efficiency for cereal production. *Agron. J.* 91 357–363. 10.2134/agronj1999.00021962009100030001x

[B121] Rodrigues C. S. Laranjo M. Oliveira S. (2006). Effect of heat and pH stress in the growth of chickpea mesorhizobia. *Curr. Microbiol.* 53 1–7. 10.1007/s00284-005-4515-8 16775779

[B122] Romeiko X. X. (2019). A comparative life cycle assessment of crop systems irrigated with the groundwater and reclaimed water in Northern China. *Sustainability* 11:2743. 10.3390/su11102743

[B123] Roy S. Liu W. Nandety R. S. Crook A. Mysore K. S. Pislariu C. I. (2020). Celebrating 20 years of genetic discoveries in legume nodulation and symbiotic nitrogen fixation. *Plant Cell* 32 15–41. 10.1105/tpc.19.00279 31649123 PMC6961631

[B124] Rubio-Sanz L. Brito B. Palacios J. (2018). Analysis of metal tolerance in *Rhizobium leguminosarum* strains isolated from an ultramafic soil. *FEMS Microbiol. Lett.* 365 1–7. 10.1093/femsle/fny010 29351606

[B125] Sablok G. Rosselli R. Seeman T. van Velzen R. Polone E. Giacomini A. (2017). Draft genome sequence of the nitrogen-fixing Rhizobium sullae type strain IS123T focusing on the key genes for symbiosis with its host Hedysarum coronarium L. *Front. Microbiol.* 8:1348. 10.3389/fmicb.2017.01348 28798728 PMC5526965

[B126] Salas A. Tortosa G. Hidalgo-García A. Delgado A. Bedmar E. J. Richardson D. J. (2020). The hemoglobin Bjgb from Bradyrhizobium diazoefficiens controls NO homeostasis in soybean nodules to protect symbiotic nitrogen fixation. *Front. Microbiol.* 10:2915. 10.3389/fmicb.2019.02915 31998252 PMC6965051

[B127] Sánchez-Cañizares C. Jorrín B. Durán D. Nadendla S. Albareda M. Rubio-Sanz L. (2018). Genomic Diversity in the endosymbiotic bacterium Rhizobium leguminosarum. *Genes* 9:60. 10.3390/genes9020060 29364862 PMC5852556

[B128] Sanchez-Lopez R. Jauregui D. Nava N. Alvarado-Affantranger X. Montiel J. Santana O. (2011). Down-regulation of SymRK correlates with a deficiency in vascular bundle development in Phaseolus vulgaris nodules. *Plant Cell Environ.* 34 2109–2121. 10.1111/j.1365-3040.2011.02408.x 21848862

[B129] Sańko-Sawczenko I. Łotocka B. Mielecki J. Rekosz-Burlaga H. Czarnocka W. (2019). Transcriptomic changes in Medicago truncatula and Lotus japonicus root nodules during drought stress. *Int. J. Mol. Sci.* 20:1204. 10.3390/ijms20051204 30857310 PMC6429210

[B130] Santos M. A. Geraldi I. O. Garcia A. A. F. Bortolatto N. Schiavon A. Hungria M. (2013). Mapping of QTLs associated with biological nitrogenfixation traits in soybean. *Hereditas* 150 17–25. 10.1111/j.1601-5223.2013.02275.x 23865962

[B131] Scott M. F. Ladejobi O. Amer S. Bentley A. R. Biernaskie J. Boden S. A. (2020). Multi-parent populations in crops: a toolbox integrating genomics and genetic mapping with breeding. *Heredity* 125 396–416. 10.1038/s41437-020-0336-6 32616877 PMC7784848

[B132] Serraj R. Sinclair T. R. Purcell L. C. (1999). Symbiotic N2 fixation response to drought. *J. Exp. Bot.* 50 143–155. 10.1093/jexbot/50.331.143

[B133] Seshadri R. Reeve W. G. Ardley J. K. Tennessen K. Woyke T. Kyrpides N. C. (2015). Discovery of novel plant interaction determinants from the genomes of 163 root nodule bacteria. *Sci. Rep.* 5:16825. 10.1038/srep16825 26584898 PMC4653623

[B134] Sessitsch A. Howieson J. Perret X. Antoun H. Martinez-Romero E. (2002). Advances in Rhizobium research. *Crit. Rev. Plant Sci.* 21 323–378. 10.1080/0735-260291044278

[B135] Shiro S. Kuranaga C. Yamamoto A. Sameshima-Saito R. Saeki Y. (2016). Temperature-dependent expression of NODC and community structure of soybean-nodulating bradyrhizobia. *Microbes Environ.* 31 27–32. 10.1264/jsme2.me15114 26877137 PMC4791112

[B136] Singleton P. W. Tavares J. W. (1986). Inoculation response of legumes in relation to the number and effectiveness of indigenous rhizobium populations. *Appl. Environ. Microbiol.* 51 1013–1018. 10.1128/aem.51.5.1013-1018.1986 16347046 PMC239003

[B137] Smýkal P. Coyne C. J. Ambrose M. J. Maxted N. Schaefer H. Blair M. W. (2015). Legume crops phylogeny and genetic diversity for science and breeding. *Crit. Rev. Plant Sci.* 34 43–104. 10.1080/07352689.2014.897904

[B138] Souza A. A. Boscariol R. L. Moon D. H. Camargo L. E. A. Tsai S. M. (2000). Effects of Phaseolus vulgaris QTL in controlling host-bacteria interactions under two levels of nitrogen fertilization. *Genet. Mole. Biol.* 23 155–161. 10.1590/s1415-47572000000100029

[B139] Sprent J. I. Ardley J. James E. K. (2017). Biogeography of nodulated legumes and their nitrogen-fixing symbionts. *New Phytol.* 215 40–56. 10.1111/nph.14474 28211601

[B140] Stambulska U. Y. Bayliak M. M. Lushchak V. I. (2018). Chromium(VI) toxicity in legume plants: modulation effects of rhizobial symbiosis. *BioMed Res. Inter.* 2018:8031213. 10.1155/2018/8031213 29662899 PMC5832134

[B141] Stanton-Geddes J. Paape T. Epstein B. Briskine R. Yoder J. Mudge J. (2013). Candidate genes and genetic architecture of symbiotic and agronomic traits revealed by whole-genome, sequence-based association genetics in *Medicago truncatula*. *PLoS One* 8:e65688. 10.1371/journal.pone.0065688 23741505 PMC3669257

[B142] Tang F. Yang S. Liu J. Gao M. Zhu H. (2014). Fine mapping of the Rj4 locus, a gene controlling nodulation specificity in soybean. *Mol. Breeding* 33 691–700. 10.1007/s11032-013-9985-y

[B143] Tang F. Yang S. Liu J. Zhu H. (2016). Rj4, a gene controlling nodulation specificity in soybeans, encodes a thaumatin-like protein, but not the one previously reported. *Plant Physiol.* 170, 26–35. 10.1104/pp.15.01661 26582727 PMC4704605

[B144] Tanya P. Srinives P. Toojinda T. Vanavichit A. Lee S. H. (2005). Identification of SSR markers associated with N2-fixation components in soybean [*Glycine max* (L.) Merr.]. *Korean J. Genet.* 27 351–359.

[B145] Thomas D. (2010). Gene–environment-wide association studies: emerging approaches. *Nat. Rev. Genet.* 11 259–272. 10.1038/nrg2764 20212493 PMC2891422

[B146] Tominaga A. Gondo T. Akashi R. Zheng S. H. Arima S. Suzuki A. (2012). Quantitative trait locus analysis of symbiotic nitrogen fixation activity in the model legume *Lotus japonicus*. *J. Plant Res.* 125 395–406. 22009016 10.1007/s10265-011-0459-1

[B147] Trněný O. Vlk D. Macková E. Matoušková M. Řepková J. Nedělník J. (2019). Allelic variants for candidate nitrogen fixation genes revealed by sequencing in red clover (*Trifolium pratense* L.). *Int. J. Mol. Sci.* 20:5470.1. 10.3390/ijms20215470 PMC6862357 31684086

[B148] Tsai S. M. Nodari R. O. Moon D. H. Camargo L. E. A. Vencovsky R. (1998). QTL mapping for nodule number and common bacterial blight in *Phaseolus vulgaris* L. *Plant Soil* 204 135–145. 10.1007/978-94-017-2321-3_13

[B149] Varshney R. K. Pandey M. K. Bohra A. Singh V. K. Thudi M. Saxena R. K. (2019). Toward the sequence-based breeding in legumes in the post-genome sequencing era. *Theor. Appl. Genet.* 132 797–816. 10.1007/s00122-018-3252-x 30560464 PMC6439141

[B150] Vitousek P. Field C. B. (2001). “Input/output balances and nitrogen limitation in terrestrial ecosystems,” in *Global Biogeochemical Cycles in the Climate System*, eds Schulze E. D. Harrison S. P. Heimann M. Holland E. A. Lloyd J. Prentice I. C. (San Diego: Academic Press), 217–225. 10.1016/b978-012631260-7/50018-2

[B151] Voung H. B. Thrall P. H. Barrett L. K. (2017). Host species and environmental variation can influence rhizobial community composition. *J. Ecol.* 105 540–548. 10.1111/1365-2745.12687

[B152] Vriezen J. A. C. De Bruijn F. J. Nüsslein K. (2007). Responses of rhizobia to desiccation in relation to osmotic stress, oxygen, and temperature. *Appl. Environ. Microbiol.* 73 3451–3459. 10.1128/aem.02991-06 17400779 PMC1932662

[B153] Wang J. Wang J. Ma C. Zhou Z. Yang D. Zheng J. (2020b). QTL Mapping and data mining to identify genes associated with the Sinorhizobium fredii HH103 T3SS effector NopD in soybean. *Front. Plant Sci.* 11:453. 10.3389/fpls.2020.00453 32508850 PMC7249737

[B154] Wang Q. Liu J. Zhu H. (2018). Genetic and molecular mechanisms underlying symbiotic specificity in legume-rhizobium interactions. *Front. Plant Sci.* 9:313. 10.3389/fpls.2018.00313 29593768 PMC5854654

[B155] Wang Y. Yang Z. Kong Y. Li X. Li W. Du H. (2020a). GmPAP12 is required for nodule development and nitrogen fixation under phosphorus starvation in soybean. *Front. Plant Sci.* 11:450. 10.3389/fpls.2020.00450 32499790 PMC7243344

[B156] Westhoek A. Field E. Rehling F. Mulley G. Webb I. Poole P. S. (2017). Policing the legume-Rhizobium symbiosis: a critical test of partner choice. *Sci. Rep.* 7:1419. 10.1038/s41598-017-01634-2 28469244 PMC5431162

[B157] Wojciechowski M. F. Lavin M. Sanderson M. J. (2004). A phylogeny of legumes (Leguminosae) based on analysis of the plastid matK gene resolves many well-supported subclades within the family. *Amer. J. Bot.* 91 1846–1862. 10.3732/ajb.91.11.1846 21652332

[B158] Yan J. Han X. Z. Ji Z. J. Li Y. Wang E. T. Xie Z. H. (2014). Abundance and diversity of soybean-nodulating rhizobia in black soil are impacted by land use and crop management. *Appl. Environ. Microbiol.* 80 5394–5402. 10.1128/AEM.01135-14 24951780 PMC4136101

[B159] Yang Q. Yang Y. Xu R. Lv H. Liao H. (2019). Genetic analysis and mapping of QTLs for soybean biological nitrogen fixation traits under varied field conditions. *Front. Plant Sci.* 10:75. 10.3389/fpls.2019.00075 30774643 PMC6367678

[B160] Yang Y. Zhao Q. Li X. Ai W. Liu D. Qi W. (2017). Characterization of genetic basis on synergistic interactions between root architecture and biological nitrogen fixation in soybean. *Front. Plant Sci.* 8:1466. 10.3389/fpls.2017.01466 PMC5572596 28878798

[B161] Yuan K. Reckling M. Ramirez M. Djedidi S. Fukuhara I. Ohyama T. (2020). Characterization of rhizobia for the improvement of soybean cultivation at cold conditions in central Europe. *Microbes Environ.* 35:ME19124. 10.1264/jsme2.ME19124 31996499 PMC7104276

[B162] Zhang B. Wang M. Sun Y. Zhao P. Liu C. Qing K. (2021). Glycine max NNL1 restricts symbiotic compatibility with widely distributed bradyrhizobia via root hair infection. *Nature Plants* 7 73–86. 10.1038/s41477-020-00832-7 33452487

[B163] Zhang F. Smith D. L. (1996). Genistein accumulation in soybean (*Glycine max* [L.] Merr.) root systems under suboptimal root zone temperatures. *J. Exp. Bot.* 47 785–792. 10.1093/jxb/47.6.785 12432039

[B164] Zhang H. Prithiviraj B. Souleimanov A. D’aoust F. Charles T. C. Driscoll B. T. (2002). The effect of temperature and genistein concentration on lipo-chitooligosaccharide (LCO) production by wild-type and mutant strains of Bradyrhizobium japonicum. *Soil Biol. Biochem.* 34 1175–1180. 10.1016/s0038-0717(02)00054-8

[B165] Zhang N. Fan X. Cui F. Zhao C. Zhang W. Zhao X. (2017). Characterization of the temporal and spatial expression of wheat (*Triticum aestivum* L.) plant height at the QTL level and their influence on yield related traits. *Theor. Appl. Genet.* 130 1235–1252. 10.1007/s00122-017-2884-6 28349175

[B166] Zhu J. Wang J. Li Q. Wang J. Liu Y. Li J. (2019). QTL analysis of nodule traits and the identification of loci interacting with the type III secretion system in soybean. *Mol. Genet. Genomics* 294 1049–1058. 10.1007/s00438-019-01553-z 30982151

[B167] Zhu X.-M. Shao X.-Y. Pei Y.-H. Guo X.-M. Li J. Song X. Y. (2018). Genetic diversity and genome-wide association study of major ear quantitative traits using high-density SNPs in maize. *Front. Plant Sci.* 9:966. 10.3389/fpls.2018.00966 30038634 PMC6046616

